# Health and quality of life outcomes impairment of quality of life in type 2 diabetes mellitus: a cross-sectional study

**DOI:** 10.1186/s12955-018-0906-y

**Published:** 2018-05-15

**Authors:** Jessie N. Zurita-Cruz, Leticia Manuel-Apolinar, María Luisa Arellano-Flores, Alejandro Gutierrez-Gonzalez, Alma Gloria Najera-Ahumada, Nelly Cisneros-González

**Affiliations:** 1 Unit of Research in Medical Nutrition, Pediatric Hospital “Centro Médico Nacional Siglo XXI”, Instituto Mexicano del Seguro Social (IMSS), Mexico City, Mexico; 2 Endocrine Research Unit, Centro Medico Nacional, Instituto Mexicano del Seguro Social (IMSS), Mexico City, Mexico; 3 Computer Research Center of Instituto Politecnico Nacional, Mexico City, Mexico; 4 Division of Prenatal Care and Family Planning Instituto Mexicano del Seguro Social (IMSS), Mexico City, Mexico; 5 Epidemiological Surveillance Coordination, Instituto Mexicano del Seguro Social (IMSS), Mexico City, Mexico

**Keywords:** Type 2 diabetes mellitus, Quality of life, Depression

## Abstract

**Background:**

Type 2 diabetes mellitus (DM2) is a chronic disease, and for treatment to succeed, it is necessary to harmonize the mental health of the patient with the environment, which impacts quality of life and adherence to medical regimens. The objetive of this study is describe the quality of life of patients with DM2 and the factors relates to its modification.

**Methods:**

This investigation was a cross-sectional study. Patients over 18 years of age with DM2 were selected. The following variables related to quality of life were studied: age, sex, occupation, marital status, years of DM2 evolution, comorbidities and presence of depression (Beck Depression Inventory). Perceived quality of life was measured with a health-related quality of life (HRQoL) scale, the 36-Item Short-Form Survey (SF-36). Patients were classified according to SF-36 HRQoL score (< 50, 51-75 and > 76 points).

**Results:**

Among the 1394 patients included, the median age was 62 years. Global HRQoL had a median of 50.1 points. Bivariate analysis showed that age, marital status, sex, occupation, comorbidities, duration of DM2 and comorbidities had impacts on HRQoL. The logistic regression model identified age (odds ratio [OR] 1.04) and depression (OR 4.4) as independent factors that influenced overall quality of life.

**Conclusions:**

Patients with DM2 have poor HRQoL, which is associated with a high frequency of depression. Older age and the presence of depression impair patient HRQoL.

**Trial registration:**

R-2013-781-052. Registered 20 December 2014.

## Background

Type 2 diabetes mellitus (DM2) is a chronic noncommunicable disease that arises when the pancreas does not produce sufficient insulin and/or when the body cannot effectively use the insulin it produces, resulting in chronic hyperglycemia [1, 2]. DM2 is directly associated with obesity, which produces peripheral insulin resistance and leads to the medium- or long-term development of DM2 [3].

In Mexico, according to the National Health Survey of 2012, there has been a significant increase in the prevalence of overweight and obesity compared to that reported in 2000, from 61.8% to 71.3% [4, 5]. Consequently, between 2000 and 2012, there was a reported increase in the prevalence of DM2 from 5.7 to 9.1% in adults 20 years of age or older, equivalent to a relative increase of almost 60% [6], and an increase in morbidity and mortality secondary to DM2.

Complications lead to increased numbers of medical appointments and hospitalizations, which affect patient quality of life (QoL) and increase the burden of hospital care costs. Macrovascular complications include systemic hypertension, acute myocardial infarction (AMI), congestive heart failure, cerebrovascular accident (CVA), and peripheral artery disease (PAD) [7]. Patients with DM2 are at a 2- to 4-fold increased risk of suffering from either AMI or CVA [8]. Microvascular complications include neuropathy, retinopathy and nephropathy [9], as well as diabetic foot syndrome [10]. These complications have an emotional and physical impact on affected individuals with DM2, causing alterations in personal and family well-being. Because of the chronic nature of the disease and the difficulty in controlling it, DM can affect mood and self-esteem, generating frustration and symptoms linked to depression; furthermore, restrictions on food and comorbidities in sexual life can lead to conflicts and contribute negatively to the QoL of the patient [11, 12].

QoL has become highly emphasized in recent years as an important health care outcome. Medicine should aim for the preservation and restoration of both the health and the dignity of the patient [13]. Consequently, it should influence not only the quantity of life but also its quality. QoL can be defined as a sense of well-being that encompasses the physical, psychological, social and spiritual condition [14]. When we refer to QoL in patients with chronic diseases, we can define it as the overall evaluation that the subject makes of his life, which depends both on the characteristics of the subject and on external factors [15].

Multiple factors have been shown to modify the QoL in patients with DM2; the most prominent factors include the presence of diabetes distress, medication adherence, depression symptomatology, longer duration of diabetes, use of insulin, marital status, and comorbidities among others [16–19].

For diabetes treatment to succeed, harmony must be achieved among the patient’s mental health, the emotional environment of the family and the control of blood glucose concentrations [20].

Although there are multiple studies where QoL has been analyzed in subjects with DM2, there are few studies conducted in developing countries, where sociocultural conditions can modify factors related to QoL, and large number of subjects and factors related to the quality of life can be assessed. Given this challenge, the objectives of our study were to describe the QoL of patients with DM2 and related factors that modify QoL.

## Methods

A cross-sectional study was performed from 1st January 2014 to 20th December 2014. The cohort included all consecutive outpatients who were over 18 years old and diagnosed with DM2, as defined by the criteria of the American Diabetes Association (ADA); patients were selected from five hospitals belonging to the Mexican Institute of Social Security (IMSS) in different cities in Mexico (Tampico, Ciudad Juárez, La Paz, Torreón and Ciudad Lerdo) [21]. Patients who knew how to read and write, had no physical limitations, and could answer the self-administered questionnaire were included. Subjects with a medical diagnosis of dementia, schizophrenia, depression or any other psychiatric diagnosis that modified the results of the questionnaires and those who did not agree to participate in the study were excluded.

Based on the results described with Zhang et al. [19], who analyzed multiple factors associated with the QoL in subjects with DM2, including age, sex, marital status, duration of the disease, comorbidities and depression, all these variables impacted the QoL; however depression presented the smallest difference in proportions, (depression being in 27.9% of subjects with adequate QoL and in 38.4% of subjects with inadequate QoL); before this, the sample size was calculated by a difference of proportions with an alpha of 5% and a power of 80%, leaving a total of 334 subjects per group, i.e., with and without depression. A total of 1894 subjects were eligible to participate; however, only 1540 patients met the inclusion criteria, of whom 146 were excluded: 13 patients had a diagnosis of dementia; 48 patients had depression and were awaiting evaluation by a specialist for initiation of pharmacological treatment; and 85 patients did not agree to participate. The 1540 patients were given the questionnaires, and upon completion, they were assessed to ensure that they were completed. Questionnaires that lacked an answer were returned to the patients to complete them.

The following variables related to QoL were studied: age; sex; occupation; marital status; years of DM2 evolution; presence of other comorbidities, such as obesity, systemic arterial hypertension, dyslipidemia and cardiac conditions; and presence of depressive symptoms. Perceived QoL was measured with a health-related quality of life (HRQoL) scale, the 36-Item Short-Form Survey (SF-36). This scale consists of 11 questions with five options and evaluates 8 scales: physical function, physical role, physical pain, general health, vitality, social function, emotional role and mental health. Possible scores range from 0 (no perceived QoL) to 100 (maximum perceived QoL). This scale has been validated in the Mexican population [22, 23].

Depressive symptoms were identified through the Beck Depression Inventory, a self-administered instrument validated for Spanish-speaking adults. Patients with depressive symptoms were defined as having a score ≥ 14. In addition to identifying patients diagnosed with depressive symptoms, we also classified the patients as having mild (score 14-19), moderate (score 20-28) or severe (score ≥ 29) depressive symptoms [24].

Patients were classified into 4 groups according to their HRQoL scores: those with scores from 0 to 25 points, from 26 to 50 points, from 51 to 75 points and from 76 to 100 points. When the results of the 1394 questionnaires administered to the patients were analyzed, none had a score lower than 25. Therefore, we ultimately analyzed them in 3 groups: Group A (inadequate HRQoL [quartile 1 and 2]), scoring 0 to 50 points; Group B (acceptable HRQoL [quartile 3]), scoring 51 to 75 points; and Group C (optimum HRQoL [quartile 4]), scoring 76 to 100 points.

In compliance with the Declaration of Helsinki, the protocol was evaluated and approved by the National Research and Health Ethics Committee of IMSS with registration number R-2013-781-052. The patients signed an informed consent letter.

### Statistical analysis

The Shapiro–Wilk test was applied to determine the distribution of the quantitative variables, and they were all found to be non-normally distributed. The variables are presented as medians, minimum (min.) values and maximum (max.) values; qualitative variables are presented as absolute numbers and percentages.

The chi-squared test and the Kruskal–Wallis test were used for comparisons among the groups with inadequate (quartile 1 and 2), acceptable (quartile 3) and optimum (quartile 4) QoL [25].

A multivariate linear regression was performed to control for confounding variables in the HRQoL scales that presented the lowest scores in the patients studied (physical function, emotional health, body pain and mental health). A multivariate logistic model was built to control for confounding variables. The association of factors with inadequate global HRQoL [26, 27] was determined using odds ratios (ORs) and 95% confidence intervals (95% CIs). All analyses were performed using SPSS version 12.0 (Chicago, IL, USA).

## Results

Of the 1394 patients included, there was no predominance with respect to sex (49.9% female, *n* = 696), and the median age was 62 years. Eighty-two percent (*n* = 1143) were married, and 41.6% (*n* = 580) of all subjects were housewives by occupation (Table 1).

**Table 1:** Comparison of demographic factor of diabetic patients by Quality of Life

		Overall sample	Quality of Life			p
			< 50	51-74	> 75	
		*n* = 1394	*n* = 690	*n* = 682	*n* = 22	
		n (%)				
Sex	Female	696 (49.9)	322 (46.7)	364 (53.4)	10 (45.5)	0.042
	Male	698 (41.1)	368 (53.3)	318 (46.6)	12 (54.5)	
Age (year)*		62 (28-77)	63 (38-77)	62 (25-76)	54 (26-78)	0.0001
Marital status	Married	1143 (82)	593 (85.9)	529 (77.6)	21 (95.5)	0.001
	Widower	109 (7.8)	52 (7.5)	57 (8.4)	–	
	Single	80 (5.8)	26 (3.8)	53 (7.8)	1 (4.5)	
	Divorced	42 (3)	15 (2.2)	27 (3.9)	–	
	Other	20 (1.4)	4 (0.6)	15 (2.3)	–	
Occupation	Housewives	580 (41.6)	298 (43.2)	275 (40.3)	7 (31.8)	0.0001
	Retired	454 (32.5)	299 (43.3)	154 (22.6)	1 (4.6)	
	Employee	205 (14.7)	37 (5.4)	157 (23)	11 (50)	
	Other	147 (10.5)	53 (7.7)	91 (13.4)	–	
	Unemployed	8 (0.5)	3 (0.4)	5 (0.7)	3 (13.6)	
Duration of diabetes(months)		240 (0-300)	252 (0-300)	132 (0-300)	72 (0-204)	0.0001
Number of morbidities	0	208 (14.9)	61 (8.8)	142 (20.8)	5 (22.7)	0.0001
	1	766 (54.9)	369 (53.5)	388 (56.9)	9 (40.9)	
	2	187 (13.4)	87 (12.6)	97 (14.2)	3 (13.7)	
	3	218 (15.7)	165 (23.9)	48 (7.1)	5 (22.7)	
	4	15 (1.1)	8 (1.2)	7 (1)	–	
Depression	No	352 (25.2)	70 (10.1)	263 (38.6)	19 (86.4)	0.0001
	Mild	223 (16)	129 (18.7)	94 (13.8)	–	
	Moderate	723 (52.9)	428 (62.1)	292 (42.8)	3 (13.6)	
	Severe	96 (6.9)	63 (9.1)	33 (4.8)	–	

"*" median (min-max)

With regard to DM2, the median duration of the disease was 240 months, and up to 85.1% (*n* = 1186) of patients presented comorbidity. Of the comorbidities recorded, the most frequent was systemic arterial hypertension (SAH), which occurred in 1044 patients (74.9%), followed by dyslipidemia in 380 patients (27.2%), obesity in 233 patients (16.7%) and cardiac conditions in 201 patients. According to the questionnaire applied, only 25.2% (*n* = 352) had no depression (Table 1).

**Fig. 1:**
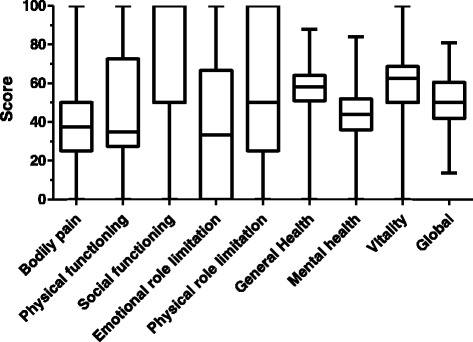
Median observed HRQoL (SF-36) dimension value

Out of a possible score of 100 points, which represents the optimum QoL, the median overall HRQoL score was 50.1 points, with a maximum of 75.5 and a minimum of 28.6 points. When analyzing HRQoL by scales, we found that physical function, emotional role, body pain and mental health had medians below 50 points, which indicates that they are the most affected scales in this group of patients (Fig. 1).

After identifying the scales with the greatest effects, we demonstrated that for physical function, the depressive symptoms, age and duration of the DM2 had negative impacts, while marital status (married) improved the score; for emotional health, the depressive symptoms, age, duration of diabetes and number of morbidities had negative impacts; for body pain, the depressive symptoms and number of morbidities had negative impacts; and for the mental health scale, the depressive symptoms, duration of diabetes and number of morbidities had negative effects. In all the scales analyzed, the depressive symptoms had significant negative effects on QoL and had the strongest effects on the physical and emotional scales (Table 2). When separating patients into groups according to the HRQoL score, we observed that almost half of the patients (49.4%, *n* = 690) had a QoL score lower than 50 points, which indicates a poor HRQoL, and only 1.5% (*n* = 22) of the patients had a score higher than 75, which indicates an optimum HRQoL.

**Table 2:** Multivariate linear regression analysis of factors associated to health-related quality of life in the modules physical function, emotional role, body pain and mental health in patients with type 2 diabetes mellitus

Factor	β	95% CI	p
PHYSICAL FUNCTION			
Depressive symptoms	−9.3	−10.5 to −8.01	> 0.001
Sex	1.78	−0.71 to 4.2	NS
Age (year)*	−0.47	−0.6 to −0.33	< 0.001
Marital status	2.86	1.65 to 4.06	< 0.001
Occupation	0.72	−0.23 to 1.68	NS
Duration of diabetes(months)	− 0.1	− 0.11 to − 0.08	< 0.001
Number of morbidities	− 0.82	−1.99 to 0.35	NS
EMOTIONAL ROLE			
Depressive symptoms	−11.6	−13.8 to −9.4	< 0.001
Sex	−1.17	−5.4 to 3.07	NS
Age (year)*	−0.72	− 0.95 to − 0.5	< 0.001
Marital status	2.13	− 0.01 to 4.17	NS
Occupation	0.43	−1.19 to 2.05	NS
Duration of diabetes(months)	−0.04	− 0.06 to − 0.02	< 0.001
Number of morbidities	−8.56	−10.5 to −6.57	< 0.001
BODY PAIN			
Depressive symptoms	−4.75	−3.44 to −6.05	< 0.001
Sex	−2.47	−5.01 to 0.05	NS
Age (year)*	0.21	−0.07 to 0.35	NS
Marital status	0.57	−0.34 to 1.8	NS
Occupation	1.59	−0.21 to 2.56	NS
Duration of diabetes(months)	−0.01	−0.03 to 0.01	NS
Number of morbidities	−5.62	−4.43 to −6.81	< 0.001
MENTAL HEALTH			
Depressive symptoms	−0.82	− 0.17 to −1.48	0.013
Sex	0.45	−0.81 to 1.71	NS
Age (year)*	0.06	−0.004 to 0.13	NS
Marital status	0.65	−0.04 to 1.26	NS
Occupation	−0.18	−0.67 to 0.29	NS
Duration of diabetes(months)	−0.01	−0.02 to − 0.01	< 0.001
Number of morbidities	−1.11	− 0.51 to − 1.7	< 0.001

"*" median (min-max)

When analyzing the factors that could influence a patient’s HRQoL group (inadequate, acceptable or optimum), we found that sex, age, marital status, occupation, duration of diabetes, number of comorbidities and depressive symptoms were statistically significant. Regarding these characteristics, Table 1 shows that the group with inadequate HRQoL (score less than 50) was older and had a greater duration of DM2, number of comorbidities, proportion of retirees and housewives, and prevalence of depressive symptoms than the group with an acceptable HRQoL (score greater than 75).

When all these variables were included in the logistic regression model, only age and depressive symptoms were identified as independent factors influencing overall HRQoL. Notably, depression (OR 4.4, 95% CI 2.03 to 9.9) had a greater impact than age (OR 1.04, 95% CI 1.0008 to 1.09) on HRQoL (Table 3).

**Table 3:** Multivariate logistic regression analysis of factors associated to bad quality life in patients with type 2 diabetes mellitus

Factor	OR	95% CI	p
Depressive symptoms	4.4	2.03-9.9	0.0001
Sex	0.75	0.29-1.94	NS
Age (year)*	1.04	1.0008-1.09	0.017
Marital status	2.06	0.93-4.4	NS
Occupation	1.11	0.77-1.6	NS
Duration of diabetes(months)	0.99	0.99-1.005	NS
Number of morbidities	0.82	0.54-1.26	NS

"*" median (min-max)

## Discussion

### Main findings of the study

In general, patients with DM2 had inadequate HRQoL, of which the most affected scales were physical function, emotional health, body pain and mental health. Depression was the factor that had the greatest impact on inadequate HRQoL.

This is one of a few recent studies investigating the population with DM2 that included a large number of subjects, in which demographic factors affecting the HRQoL were associated (multivariate analysis); in addition, it did not show that sex affected global HRQoL or physical function, emotional health, body pain and mental health [16, 28].

The determination that patients with DM2 present low HRQoL coincides with previously published results in which DM2 had a negative impact on QoL, mediated by factors such as the need for a strict dietary plan, exercise and a specific treatment regimen [29, 30]. The findings in the literature regarding the QoL of patients with DM2 and its association with sociodemographic factors have been variable. Previous reports found that lower educational level, lower income and belonging to the female sex were associated with poor QoL in people with diabetes [31].

The identified factors impacting QoL, such as older age and depression, impact glycemic control, which could be an added factor that deteriorates QoL [32]. Another important factor is that patients with DM2 often feel challenged by their illness and the related demands on a daily basis, which also impacts their perception of QoL [33].

Several studies have shown that the presence of comorbidities decreases the QoL of patients with diabetes; for example, Wermeling et al. evaluated 2086 patients with DM2 in the Netherlands and found that those with comorbidities had a significantly lower health status than those without comorbidities [34]. In contrast, a study conducted in Singapore failed to find such an association [35]. Factors such as the time course of diabetes and the use of insulin have also been negatively associated with QoL. In the present study, 85.1% of patients with DM2 presented at least one non-psychiatric medical comorbidity; however, in the multivariate analysis, these comorbidities were not found to impact QoL. Although we did not observe an effect, it is important that health care providers take special care in managing the comorbidities of DM2, as other studies have shown that QoL worsens and that survival drastically decreases as the number of comorbidities increases [36].

Furthermore, the results of the present study suggest that depression is common among patients with DM2 and is associated with the perception of a poor QoL; depression should be screened for in these patients, especially older patients, who face greater risks related to the lack of motivation and emotional exhaustion [19, 37].

Depression and diabetes interact so closely that it is difficult to identify which pathology begins first; the diagnosis of DM2 causes mourning for the loss of health, which favors the evolution of depression, and a depressed state can promote poor eating habits [38]; that is, depression interferes with the ability to initiate healthy life patterns and mitigate risk at the onset of DM2. Emphasis should be placed on the need to better understand any overlap between depression and difficulty in adhering to pharmacological treatment and changes in lifestyle.

The psychological and pharmacological treatment of depression in subjects with diabetes is associated with significant clinical improvements. Such improvements occur not only in mood but also in adherence to diet and treatment regimens for DM2, thereby impacting glycemic control, reducing chronic complications and improving QoL [38].

The high incidence of elevated depressive symptoms in the sample may be because depressed patients have an increased risk not only for diabetes but also for metabolic syndrome, which is sometimes defined as pre-diabetes [39]. Therefore, it is possible that there is a bidirectional association between depression and diabetes, i.e., depression can increase the risk for metabolic risk factors that subsequently develop into DM, which increases the risk for mental health status impairment and poor QoL.

Management of DM2 is complicated by psychosocial challenges, and it is important to recognize the potential influence of depression and the deterioration of QoL in the prognosis and management of the disease, as evidenced by the results of this and other studies [38, 40–42]. Additionally, for better control and monitoring of the disease, a combination of effective activities should be implemented to improve self-care [40].

Consequently, identifying the epidemiological profile and factors associated with QoL in these patients will contribute to the design of a comprehensive program that includes interventions for effective self-care, promotion of the correct use of medicines and promotion of healthy individual and collective conditions.

However, depression is not a DM-specific risk factor for impaired QoL as it is reproducible across different patient populations. Depression was documented as an important predictor of impaired QoL across non-DM patient populations, such as patients with brain tumors and older persons [43, 44].

One limitation of the study was the type of design, which, being a transversal study, could not identify the relationship between depression and poor perceived QoL, and we could not ascertain which came first. In addition, the study did not analyze QoL according to the presence of chronic complications secondary to DM2.

Depression has an important impact on HRQoL in patients with DM2, and thus, strategies should be developed to prevent depression. It has been shown that exercise on a regular basis, of any type of intensity, prevents depression [45, 46]. Because of this, some of the strategies should be to promote routine exercise for older adults.

## Conclusions

In general, patients with DM2 have inadequate QoL. The factors related to QoL include depression, and older patients with depression showed a greater deterioration in HRQoL.

The results of this study indicate that it is essential to support DM2 patients by implementing integral management strategies with support groups.

## Abbreviations

ADA: American Diabetes Association
AMI: Acute myocardial infarction
CHF: Congestive heart failure
CVA: Cerebrovascular accident
DM2: Type 2 diabetes mellitus
HRQoL: Health-related quality of life
IMSS: Instituto Mexicano del Seguro Social
PAD: Peripheral artery disease
QoL: Quality of Life
